# The *YABBY* Genes of Leaf and Leaf-Like Organ Polarity in Leafless Plant *Monotropa hypopitys*

**DOI:** 10.1155/2018/7203469

**Published:** 2018-04-24

**Authors:** Anna V. Shchennikova, Marya A. Slugina, Alexey V. Beletsky, Mikhail A. Filyushin, Andrey A. Mardanov, Olga A. Shulga, Elena Z. Kochieva, Nikolay V. Ravin, Konstantin G. Skryabin

**Affiliations:** ^1^Federal State Institution “Federal Research Centre “Fundamentals of Biotechnology” of the Russian Academy of Sciences”, Moscow 119071, Russia; ^2^Lomonosov Moscow State University, Moscow 119991, Russia

## Abstract

*Monotropa hypopitys* is a mycoheterotrophic, nonphotosynthetic plant acquiring nutrients from the roots of autotrophic trees through mycorrhizal symbiosis, and, similar to other extant plants, forming asymmetrical lateral organs during development. The members of the YABBY family of transcription factors are important players in the establishment of leaf and leaf-like organ polarity in plants. This is the first report on the identification of *YABBY* genes in a mycoheterotrophic plant devoid of aboveground vegetative organs. Seven *M. hypopitys YABBY* members were identified and classified into four clades. By structural analysis of putative encoded proteins, we confirmed the presence of YABBY-defining conserved domains and identified novel clade-specific motifs. Transcriptomic and qRT-PCR analyses of different tissues revealed *MhyYABBY* transcriptional patterns, which were similar to those of orthologous *YABBY* genes from other angiosperms. These data should contribute to the understanding of the role of the *YABBY* genes in the regulation of developmental and physiological processes in achlorophyllous leafless plants.

## 1. Introduction

*Monotropa hypopitys* (syn. *Hypopitys monotropa*) is a member of the flowering seed plant family Ericaceae, which in turn belongs to the order Ericales splitting from the base of the clade Asterids [1]. This mycoheterotrophic, nonphotosynthetic, achlorophyllous plant acquires carbon from the roots of autotrophic trees through monotropoid mycorrhizal symbiosis [2, 3]. The *M. hypopitys* root system consists of fleshy roots, on which shoot buds develop, and finer mycorrhizal roots [2]. This plant has a typical aboveground structure, although the stem and leaves can be taken for flowering parts—floral axis and sterile bracts [4, 5]. Similar to extant plants, *M. hypopitys* forms asymmetrical lateral organs on the flanks of a shoot or inflorescence apical meristem, with adaxial and abaxial surfaces adjacent to or distant from, respectively, the meristem. Paleobotanic studies indicate that such structural asymmetry first appeared in a true leaf (euphyll) transformed from a radially symmetric stem as a consequence of the need to absorb more sunlight [6–9]. Studies of asymmetry in plants indicate that during plant development, cell fate is determined mostly by positional signals. The correct maintenance of the apical meristem and abaxial-adaxial differentiation of lateral organs requires reciprocal signal interaction between the meristem and derived structures [10–12].

It has been shown that the polarity of leaves and floral organs is defined by the network of genes encoding the Class III Homeodomain Leucine Zipper (HD-ZIPIII), ASYMMETRIC LEAVES (AS1/AS2), KANADI (KAN), AUXIN RESPONSE FACTOR (ARF3/ARF4), and YABBY families of transcription factors [8, 13–15]. Among them, the HD-ZIPIII REVOLUTA (REV) is expressed in the adaxial domain of lateral organs, whereas the GARP-family transcription factors KAN1–4 are involved in abaxial differentiation. Together, REV and KAN1 antagonistically regulate the expression of a number of genes encoding auxin signaling and transport components [16, 17].

The *YABBY* genes originating from the lineage leading to seed plants are identified in various green spermatophyte plant species; they are closely associated with the evolutionary emergence of flat-shaped leaves and are presumably diversified during evolution, which resulted in the appearance of family members with specific functions in the leaf, carpel, and ovule [8, 18–20]. YABBY transcription factors are characterized by their nuclear localization and the presence of the C_2_C_2_ zinc-finger and DNA-binding YABBY (High Mobility Group- (HMG-) box-like) domains [21, 22]. In gymnosperms, the *YABBY* genes are distributed among the A, B, C, and D clades [23]. In extant angiosperms, five *YABBY* subfamilies, *FILAMENTOUS FLOWER (FIL* or *YAB1)*/*YABBY3 (YAB3* or *AFO)*, *CRABS CLAW (CRC)*, *INNER NO OUTER (INO* or *YAB4)*, *YAB2*, and *YAB5* [21] are distinguished by conserved functions in the initiation of lamina outgrowth, polarity maintenance, and establishment of the leaf margin [23–25]. Almeida et al. [26] and Morioka et al. [27] have provided evidence for the involvement of the *YAB2* and *YAB5* genes in the evolutionary diversification of style and filament morphology. The branching of *INO* and *CRC* from other *YABBY* genes has most likely occurred in parallel with the evolution of the carpel and outer integument via modification of reproductive leaf-like sporophylls [20, 28].

There are current theories related to the history of the *YABBY* genes in angiosperms. Bartholmes et al. [29] suggested that “vegetative” *YABBY*s *(FIL*/*YAB3*, *YAB2* and *YAB5)* do not form a monophyletic clade and that *CRC* and *FIL* evolved from a common ancestor gene, while the *INO* genes are sisters to that ancestral gene. On the other hand, Finet et al. [23] clustered *INO* together with clades *YAB5* and *YAB2*. In addition, two alternative evolutionary scenarios, that is, monophyly or paraphyly of the gymnosperm *YABBY* family towards angiosperm *YABBY* genes, suggest that all spermatophyte *YABBY* genes were derived from one or two, respectively, *YABBY* genes of the last common ancestor of extant seed plants [20, 23, 29]. The reconstruction of *YABBY* evolution in spermatophytes based on these theories suggests that at least one *YABBY* predecessor has already functioned as a polarity regulator and that the diversification of the gene family occurred in both angiosperms and gymnosperms [23]. Although the presence of the YABBY genes is presumably restricted to seed plants [19, 20], genomics studies conducted on marine picoeukaryotes revealed *YABBY* homologs in Chlorophyta [30]. Phylogenetic analysis of identified sequences suggested, with equal probability that either Chlorophyta *YAB* genes are evolutionarily related to seed plant *YABBY*s or emerged independently from ancestral HMG-box sequences [23].

The expression data available for the angiosperm *YABBY* genes suggested that the *FIL*-, *YAB2*-, and *YAB5*-like genes retained a more ancestral expression pattern in both vegetative and reproductive tissues, while the expression of the *CRC* and *INO*-like genes is more variable [29]. In Eudicots, *FIL*, *YAB3*, *YAB2*, and *YAB5* transcripts were detected in the abaxial side of primordia in all aboveground lateral organs (except ovules) determining the abaxial cell fate [31, 32]. The *CRC* genes are expressed abaxially in the carpel, placenta, and nectaries promoting the development of the gynoecium and abaxial part of the carpel wall, and terminating the floral meristem [33–38]. *INO* mRNA is detected in the abaxial epidermis of the outer integument [20, 28, 39, 40]. The *YABBY* expression pattern differences between cereal monocots and other angiosperms indicate the modification of genetic pathways involving *YABBY*s during the process of angiosperm diversification [21, 27, 41–46]. It is assumed that FIL, together with REVOLUTA (REV), APETALA1 (AP1), and LEAFY (LFY), corrects the spatial activity of the *AGAMOUS (AG)*, *AP3*, *PISTILLATA (PI)*, and *SUPERMAN (SUP)* genes, and, thus, is involved in the initiation of floral organ primordia at the correct position and numbers, defining the fate of appropriate cells [31, 44, 47].

Thus, the bifunctional YABBY transcription factors have an important role in driving the evolution of the leaf and gynoecium, as well as in the initiation, growth, and structural organization of almost all aboveground lateral organs, and in the control of shoot apical meristem organization and activity.

In the present study, we identified and phylogenetically classified seven *YABBY* members from *M. hypopitys* and characterized their expression profiles in various tissues during flowering. The structural features and composition of conserved motifs belonging to the predicted MhyYABBY proteins were also analyzed. Our data should further the understanding of possible links between polarity determination and the physiology of achlorophyllous mycoheterotrophic plants.

## 2. Materials and Methods

### 2.1. Plants and Transcriptomes

The previous study divided *M. hypopitys* specimens into a North American cluster and two Eurasian (excluding Russia) sister lineages (Swedish and pan-Eurasian) [48]. Analysis of *M. hypopitys* from the European part of Russia revealed two types, A and B, which showed 99 and 100% homology with specimens from Japan, Finland, and Great Britain, and with Swedish specimens, respectively [49–51]. The study of *H. monotropa* specimens from Northern Ireland showed that they occur in small, highly fragmented populations, and exhibit a relatively high level of within-population genetic diversity and a low level of clonality [52].

In the present study, two *M. hypopitys* plants of type B from one clone of the same genet were used. Flowering plants were collected in a coniferous forest, Kaluga region, Russia, in August, 2015. The individual plant was a 15 cm reproductive axis with bracts and raceme of 10–12 flowers (each of 4 sepals, 4 petals, 8 stamens, and 4 fused carpels), and root system comprised mycorrhizal and fleshy roots with adventitious buds. The annual floral axes arise from adventitious buds on the perennial roots and carry the laminar appendages (there are no flowers in their axils, but they are above the soil level), which are termed sterile bracts (in our study, bracts, for simplicity) [4].

Plants were dissected into flowers, bracts, fleshy roots with adventitious buds, and predominantly haustoria-enriched roots, immediately frozen and homogenized in liquid nitrogen, and stored at −80°C. Total RNA was isolated from tissue of each *M. hypopitys* bracts (two individual plants), flowers (two individual plants), roots containing buds (individual plant), and haustoria-enriched roots (individual plant) and used for mRNA library preparation, which was sequenced on the Illumina HiSeq2500 platform (Illumina Inc., San Diego, CA, USA). The *M. hypopitys* RNA-seq data for each of six transcriptomes were assembled into the 98,350 unigenes with a length of 201–12,993 bp [51, 53]. Individual reads were mapped on contigs using Bowtie 2 [54], and protein-coding genes in contigs were identified using TransDecoder (https://transdecoder.github.io/).

### 2.2. Identification and Bioinformatics Characterization of *M. hypopitys* YABBY-Coding Sequences

To identify the *M. hypopitys* genes homologous to the known organ polarity genes, we searched unique transcripts revealed by the RNA-seq against the NCBI database (http://blast.ncbi.nlm.nih.gov/). To predict *YABBY* transcripts in *M. hypopitys*, we additionally searched the assembled transcriptomes with the known *YABBY*-related sequences coding for conserved zinc-finger and HMG-like domains extracted from the NCBI database. Selected *YABBY* candidates were examined for open reading frames (ORFs), translated using Clone Manager v.7.11 (http://clone-manager-professional.software.informer.com/), and the conserved domains of putative MhyYABBY proteins were identified using the NCBI-CDD analyzer (http://www.ncbi.nlm.nih.gov/Structure/cdd/wrpsb.cgi) and specified according to [21].

To evaluate the overlap between transcriptomes, Venn diagrams were generated using the online program Venny [55]. To illustrate the transcriptome-based gene expression pattern, the data were clustered with the Average linkage method and Spearman rank correlation (distance measurement method), and visualized as a heat map (http://www2.heatmapper.ca/) [56].

Conserved MhyYABBY amino acid motifs were identified using the MEME (Multiple Expectation Maximization for Motif Elicitation) 4.11.2 online analysis (http://meme-suite.org/tools/meme) [57] and used to construct a schematic diagram. To search for motifs, the “Normal” motif discovery mode, the default width range of 6–50 amino acids (aa), and the motif site distribution “zero or one per sequence” were used. The identified motifs were manually compared with previously suggested specific motifs. Initial search was performed on a set of 34 complete sequences, including identified MhyYABBYs, independently of YABBY clade affiliation. In addition, variable regions between conserved domains within the same group of proteins were searched. Since most of the identified motifs were clade-specific, we further analyzed individual YABBY clades.

To investigate the evolutionary relationship of the *MhyYABBY* genes, the MhyYABBY proteins and YABBY homologs from other species available in NCBI were aligned using ClustalX [58]. For analysis, full-size amino acid sequences, as well as conserved regions consisting of the zinc-finger and HMG-like domains were used. Evolutionary divergence between the genes and proteins was estimated using the maximum composite likelihood and equal input models, respectively, in MEGA7 [59–62]. The phylogenetic tree topology was estimated using the maximal likelihood method based on the JTT matrix-based model in MEGA7 [62].

### 2.3. Analysis of Tissue-Specific Gene Expression

The *MhyYABBY* gene expression was calculated in each transcriptome. Transcript quantification based on RNA-seq data was performed without a reference genome using the RSEM [63] and Bowtie 2 [54] programs, including normalization of transcripts per kilobase of exon per million fragments mapped (FPKM) values and between samples.

To perform quantitative real-time PCR (qRT-PCR), the first strand cDNA was synthesized from 1 *μ*g of each mixture of two-root, two-bract, and two-flower RNA preparations using the Reverse Transcription System (Promega, Madison, WI, USA) and an oligo-dT primer, and quantified using the Qubit® Fluorometer.

Based on the identified *YABBY*-like transcripts and corresponding draft genomic sequences (our unpublished data), gene-specific primers separated by at least one big intron were designed to amplify parts of gene-coding sequences (Supplementary Table 1). The qRT-PCR was performed in three technical replicates using 2.5 ng of cDNA and an SYBR Green and ROX RT-PCR mixture (Syntol, Moscow, Russia) at the following cycling conditions: initial denaturation at 95°C for 5 min, 40 cycles of denaturation at 95°C for 15 sec, and annealing/synthesis at 60°C for 40 sec. Obtained PCR-fragments were additionally purified and sequenced to confirm certain gene specificities. Gene expression levels were normalized to those of the reference pinesap *Actin5*, *Actin3*, and *SAND* genes (primers are provided in Supplementary Table 1), which transcripts were evenly represented in six transcriptomes [51]. Normalized expression data were statistically evaluated using GraphPad Prism version 7.02 (San Diego, CA, USA; https://www.graphpad.com/scientific-software/prism/). Three values (3 technical replicates) were used for SD calculation. The error bars were generated based on mean with SD calculation. Significance of the qRT-PCR data within the same tissue between species was estimated by unequal variance Welch's *t*-test and additionally treated with Bonferroni's correction: if any of the *t*-tests in the list had *p* ≤ 0.05/number of *t*-tests in the list, then the null hypothesis was rejected; that is, the difference between samples was recognized as significant.

## 3. Results

### 3.1. *M. hypopitys* Organ-Polarity Genes

**Figure 1:**
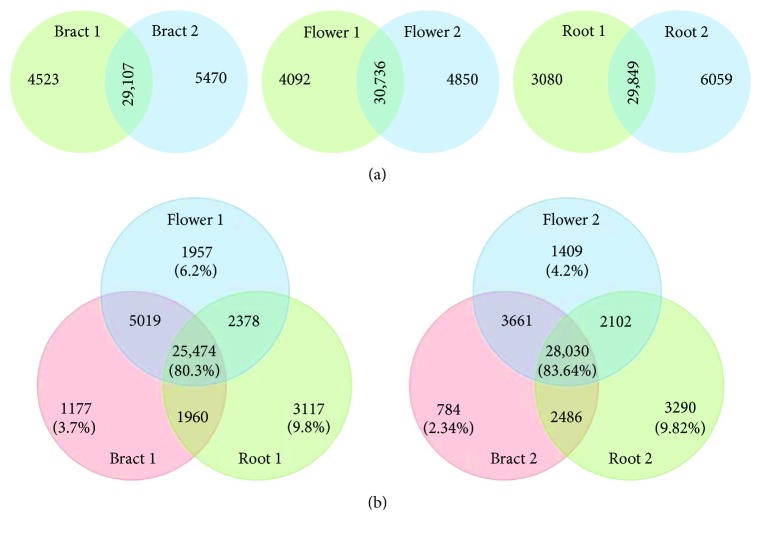
Venn diagrams of the overlap between the whole set of reads in paired transcriptomes (a) and in three transcriptomes for each of the two plants analyzed (b).

**Figure 2:**
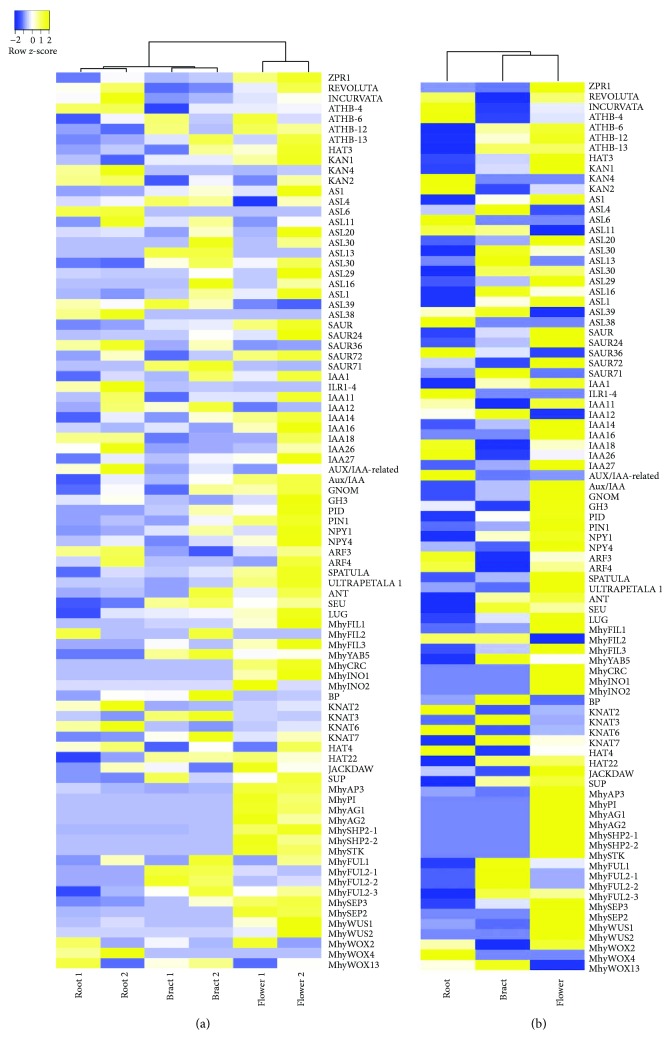
Heat maps of the organ polarity gene expression in six transcriptomes (a) and the mean expression of these genes in paired transcriptomes (b).

The obtained six transcriptomes [53] showed significant overlap between the whole set of reads of paired libraries (74–77%), and in three libraries for each of the two plants (>80%) (Figure 1), and reflected a number of the known plant organ polarity genes that are being expressed in different tissues of flowering pinesap (Supplementary Table 2). Among them, the genes of the KAN and REV transcription factors, responsible for the abaxial and adaxial cell identity in lateral organs, respectively, and their common targets *(ARF*, *SAUR*, *Aux*/*IAA*, *PID*, *PIN*, *NPY*, *GH3*, etc.) involved in auxin biology [17] were identified. The genes encoding SEUSS (SEU) and LEUNIG (LUG) involved in petal polarity determination along the adaxial/abaxial axis [64], AP2-like transcription factor AINTEGUMENTA (ANT) which contributes to organ polarity [65], the ADP ribosylation factor guanine nucleotide exchange factor GNOM essential for basal polarity establishment in *A. thaliana* [66], ULTRAPETALA1 (ULT1) which acts antagonistically with KAN1 to pattern the adaxial-abaxial polarity axis but jointly to pattern the apical-basal axis restricting the expression domain of the *SPATULA* gene [67], and HD-ZIPIII transcription factors HAT and ATHB positively regulated by REV [68] were also found (Supplementary Table 2). Heat map-based clustering of transcriptomic reads associated with organ polarity genes revealed similarities between pair libraries (according to the column dendrogram in Figure 2(a)). For most genes, the expression levels in flowers were higher than those in bracts (Figure 2(b)).

**Figure 3:**
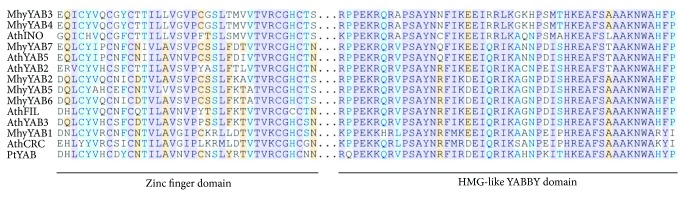
Alignment of conserved zinc-finger and HMG-like domains from putative *M. hypopitys* YABBY proteins 1–7, known as *A. thaliana* YABBYs (AthINO, AF195047; AthCRC, AF132606; AthFIL, AF136538; AthYAB3, AF136540; AthYAB5, NP_850081; AthYAB2, AF136539), and *Pinus taeda* PtYAB (DR100835; gymnosperms).

All six transcriptomes of bracts, flowers and roots contained seven unique *YABBY-*like transcripts, which were considered as putative *MhyYABBY*s *(MhyYAB1*–*MhyYAB7)*. The size of putative *MhyYABBY* ORFs varied from 502 to 673 bp, and the length of predicted proteins was from 166 to 224 aa. One transcript, *MhyYAB4*, had a partial 3′-truncated coding sequence (CDS), while the remaining six mRNAs contained complete CDSs. Putative MhyYABBY proteins included both a conserved N-terminal 37-aa C_2_C_2_ zinc finger-like domain and a C-terminal 48-aa helix-loop-helix domain resembling a part of an HMG box (Figure 3) [21, 33]. A cluster of amino acids at the beginning of the HMG-like domain could potentially serve as a nuclear localization signal [33, 69].

Among *MhyYABBY*s, we distinguished two groups (comprising *MhyYAB2*, *MhyYAB5*, and *MhyYAB6*; and *MhyYAB3* and *MhyYAB4*) based on high identity outside the conserved domains (Supplementary Figures 1a, 1b). The estimated low evolutionary pairwise divergence in the nucleotide and amino acid sequences between the members of each group compared to that between the members of different groups suggested the presence of two sets of paralogs (Supplementary Tables 4 and 5). *MhyYAB1* showed high pairwise divergence with other *MhyYABBY*s, which indicates the affiliation of *MhyYAB1* to the separate clade.

### 3.2. Phylogeny of the *M. hypopitys YABBY* Family

Previous phylogenetic studies considered only conserved YABBY domains; however, it has also been shown that variable protein regions contain clade-specific conserved motifs of potential functional importance [29]. In transcription factors, variable regions are often essential for their activity and/or formation of multimeric protein complexes, as it has been shown for MADS-box transcription factors [70]. We aligned complete amino acid sequences and only conserved domains of MhyYABBYs. For the group comprising MhyYAB2, MhyYAB5, and MhyYAB6 paralogs, two different results within the same clade were obtained. The full-size proteins were orthologous to FIL, while the conserved domains were orthologous to YAB3, another member of the FIL/YAB3 clade. We decided to use complete sequences to increase the sensitivity of phylogenetic analysis.

**Figure 4:**
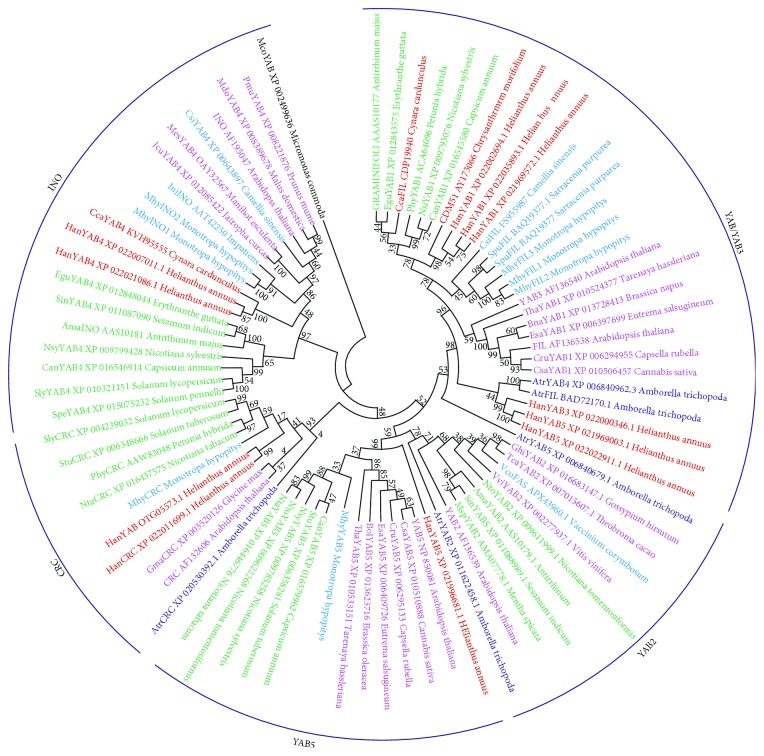
A phylogenetic tree of MhyYABBY proteins was generated with 83 full-size YABBY-like amino acid sequences from *M. hypopitys*, *A. thaliana*, and other angiosperm species. The *M. commode* (Chlorophyta) YABBY-like protein was used as outgroup. The *Amborella trichopoda*, Ericales, Rosids, Lamiids (euAsterids), and Campanulids (euAsterids) sequences are colored in dark blue, blue, burgundy, green, and red, respectively. The numbers next to the nodes represent bootstrap values from 1000 replicates.

The generated tree, rooted with the *Micromonas commode* (Chlorophyta) YABBY-like protein, classified MhyYABBY1–7 by comparing them with YABBY-like proteins of *A. thaliana* and other angiosperm species belonging to Asterids and Rosids. As a result, *MhyYABBY* transcripts were distributed into the *FIL*, *INO*, *CRC*, and *YAB5* clades and renamed accordingly as *MhyCRC (MhyYAB1)*, *MhyINO1* and *MhyINO2 (MhyYAB3* and *MhyYAB4*, resp.), *MhyYAB5 (MhyYAB7)*, and *MhyFIL1*, *MhyFIL2*, and *MhyFIL3 (MhyYAB5*, *MhyYAB6*, and *MhyYAB2*, resp.). It should be noted that no *YABBY2*-like transcripts were found in all six transcriptomes. The sequences have been deposited in GenBank (KX12839–KX12841, KX12843–KX12846; Supplementary Table 3). The maximum likelihood reconstruction of the MhyYABBY family with bootstrap values at tree nodes is shown in Figure 4. Within the individual clades, MhyYABBY sequences are sisters to other YABBY-like sequences from Asterids, and the closest homologs are YABBYs from Ericales.

### 3.3. MhyYABBY-Specific Motifs Identified outside of the YABBY Domains

To further investigate the structural divergence of pinesap YABBY proteins, we searched for the motifs conserved within individual clades or the whole MhyYABBY group. Comparison of complete sequences of YABBY orthologs (Supplementary Table 6) revealed two major domains, zinc-finger and YABBY, specific to all YABBY proteins. The number of motifs in variable regions was 4 to 9 with the length from 5 to 26 residues. The obtained data were compared with previously defined motifs specific to individual YABBY clades [29].

In MhyINO1/2, the known INO-A motif was found immediately after the zinc-finger domain. Comparison of MhyINO1/2 with other INO-like sequences in the NCBI database revealed novel putative INO-specific motifs. At the N-terminus and between the zinc-finger and YABBY domains, a highly conserved 11-aa INO-B motif and an Eudicot-specific 15-aa INO-C motif were identified. In addition, we found a C-terminal 26-aa INO-D motif specific to MhyINO1/2 and INO-like proteins in Solanaceae. All previously predicted CRC-specific motifs, CRC-A, CRC-B, CRC-C, and CRC-D, were found in MhyCRC. In addition, we identified a putative conserved Eudicot-specific C-terminal 15-aa CRC-E motif. In MhyYAB5, the presence of the known YAB5-specific motifs YAB5-A, GY/YAB2/5-A, YAB5-B, and GY/YAB2/5-B was confirmed. The YAB5-D motif was not detected, while a modified YAB5-C sequence was identified before the YABBY domain as a 12-aa YAB5-Cm motif. In MhyFIL1/2/3, we found all previously predicted motifs, FIL-A, FIL-B, FIL-C, FIL-D, FIL-E, FIL-F, and FIL-G. In addition, we suggested two motifs, 12-aa FIL-I (monocot- and eudicot-specific) and 11-aa FIL-H (eudicot-specific) located between the zinc-finger and YABBY domains, as candidates for conserved FIL-specific motifs.

**Figure 5:**
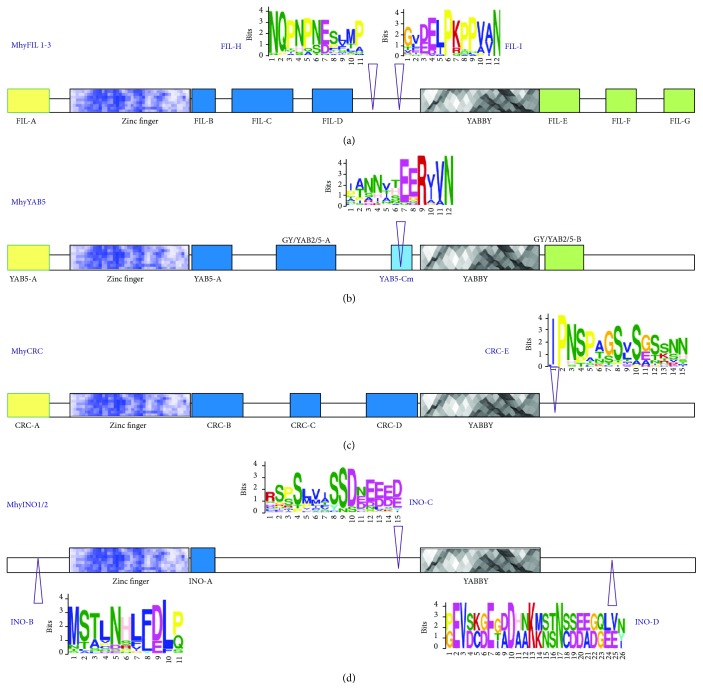
Amino acid clade-specific motifs predicted in *M. hypopitys* YABBY proteins. MhyFIL1/2/3 (a); MhyYAB5 (b); MhyCRC (c); MhyINO1/2 (d). Previously suggested clade-specific motifs [29] are shown as boxes. MEME-predicted novel motifs are marked by arrowheads and shown as letter sequences.

Thus, we characterized MhyYABBY proteins by sequence conservation on the clade level. The motifs identified in MhyYABBYs were shown in Figure 5 and sequences of the predicted novel motifs are presented in Supplementary Figure 2).

### 3.4. Expression Pattern of “Vegetative” and Flower-Specific *M. hypopitys YABBY* Genes

**Figure 6:**
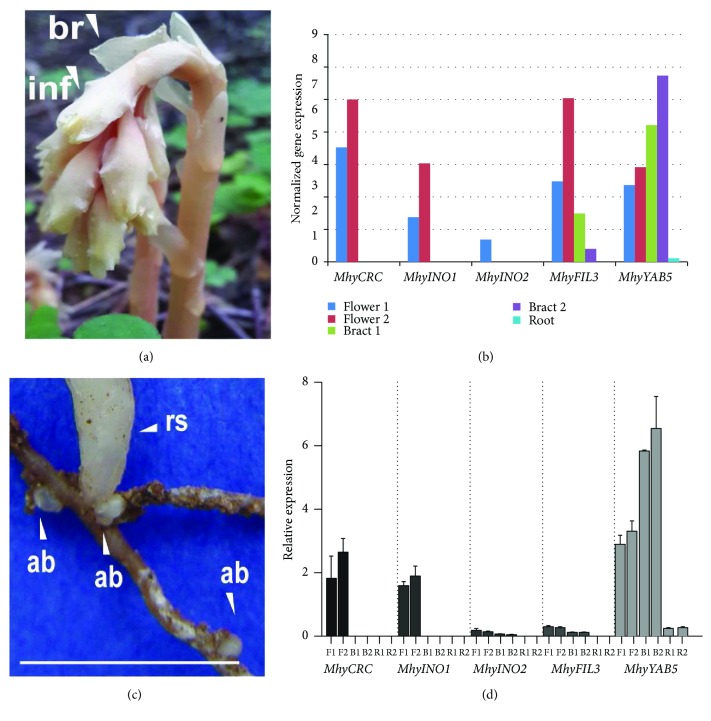
Expression profiles of *M. hypopitys YABBY* genes in the bract, flower, root and bud, and haustoria-enriched root tissues. Expression was estimated as the transcript number per million equal to the sum of total transcripts in the tissue. The *M. hypopitys* adult plant; br—bract, fl—flower (a); transcriptome-based expression pattern of the *MhyFIL1*, *MhyFIL2*, *MhyFIL3*, *MhyYAB5*, *MhyCRC*, *MhyINO1*, and *MhyINO2* genes in pinesap tissues (b); the *M. hypopitys* roots with adventitious buds; ab—adventitious bud, rs—growing reproductive stem; scale bar = 1 cm (c); relative expression (qRT-PCR) of the pattern of the *MhyCRC*, *MhyINO1*, *MhyINO2*, *MhyFIL3*, and *MhyYAB5* genes in pinesap tissues, F—flower, B—bract, R—root (d).

The transcriptome-based data on *MhyYABBY* expression in the roots and buds, haustoria-enriched roots, bracts, and flowers (Figure 6, Supplementary Table 3) showed that the *MhyYABBY* mRNAs (except *MhyFIL2*) were present in the flowers. Except for *INO*- and *CRC*-like *MhyYABBY*s, all other five transcripts were detected in the bracts. The highest bract-specific expression was observed for *MhyYAB5*, while the remaining four genes were transcribed at similarly low levels. Finally, in roots and buds, only *MhyYAB5* and *MhyFIL2* mRNAs were expressed at very low levels, while in the haustoria-enriched roots none of genes were expressed. The *MhyFIL1* and *MhyFIL3* transcripts were increased from the bract to the flower, maintaining the same expression profile. In contrast with this, the number of *MhyYAB5* transcripts was decreased from the bract to the flower.

Quantitative (q) RT-PCR data on *MhyFIL3*, *MhyYAB5*, *MhyINO1*, *MhyINO2*, and *MhyCRC* expression are represented at Figure 6(d) and Supplementary Table 7. The relative expression of *MhyYABBY* genes was estimated in the flowers, bracts, and roots and buds. All analyzed genes were expressed in the flowers with the highest *MhyYAB5* level, and the lowest *MhyINO2* and *MhyFIL3* levels. In the bracts, the *MhyYAB5* gene was also highly expressed, but only traces of the *MhyINO2* and *MhyFIL3* mRNAs were observed. In the roots and buds, the only *MhyYAB5* mRNA was detected at low level. The difference in the *MhyYAB5* gene expression between the pinesap tissues was statistically significant. The flower-specific expression of *MhyCRC* and *MhyINO1* was significantly different from that in bracts and roots. The expression of *MhyINO2* and *MhyFIL3* was similar between tissues (Supplementary Table 7). All the analyzed gene expression modes were the same as it was shown in their transcriptome-based patterns, except for *MhyINO2*. In the flower, the measured qRT-PCR expression of this gene was equally low. Given transcriptomic data, *MhyINO2* was absent in flower 2, which may be due to the quality of the libraries or their sequencing. Also, considering the low evolutionary pairwise divergence in the *INO1* and *INO2* sequences, a higher level of *INO1* expression compared to the level of *INO2* may indicate that *INO1* may be more required than *INO2* during plant development.

## 4. Discussion

The emergence of photosynthesis has become the most significant event in the evolution of plants. The majority of extant plants are autotrophic, except for about 1% of flowering heterotrophic plants. Among the latter, obligate mycoheterotrophs are the results of replicated deevolutionary events of the photosynthetic ability loss, triggering the degradation of both cytoplasmic and nuclear genomes [71]. Full mycoheterotrophs demonstrate a wide range of deevolutionary outcomes such as abrupt morphophysiological changes [2], genome rearrangements, and massive gene loss [71, 72].

In the large and diverse eudicot family Ericaceae with a nearly worldwide distribution, two of nine subfamilies, Pyroloideae and Monotropoideae, contain partial and full mycoheterotrophs, respectively [73]. In Monotropoideae, *M. hypopitys* represents a unique obligate mycoheterotroph. Recent studies on the *M. hypopitys* plastid genome and its comparison with that of photosynthetic relative *Pyrola rotundifolia* indicated that this plant is at the final stages of plastome degradation, which is expressed in highly reduced size and content, dramatic structural rearrangements, and acceleration of nucleotide substitutions in all protein-coding genes [74–76]. Furthermore, the coordinated loss of photosynthesis-related functions in both plastome and nuclear genomes of *M. hypopitys* is a sign of ongoing changes in the nuclear genome of this mycoheterotrophic plant [51].

It is generally accepted that mycoheterotrophic plants have evolved from photosynthetic mycorrhizal lineages, as mycoheterotrophy helps to succeed in the low-light conditions of the forest [77]. It has been established that dark-induced leaf senescence leads to a significant chlorophyll loss and photosynthesis inactivation [78, 79]. During evolution, a *M. hypopitys* ancestor (already with megaphylls) growing in shaded habitats lost the genetic ability to photosynthesize due to symbiosis with fungi, which provided a sufficient amount of carbon to pinesap from the roots of autotrophic trees. It is shown that the loss of photosynthetic ability and full heterotrophy are linked to the degradation and/or modification of vegetative structures [77]. It is believed that in *M. hypopitys*, an elongated raceme emerges instead of a true stem, developing directly from the adventitious bud on the roots [4].

The photosynthetic ability is closely related to the origin of asymmetrical leaves (providing the absorption of sufficient light energy by seed plants [80, 81]), in particular, due to the *YABBY* genes' evolutionary duplication and diversification [6–9, 25, 82, 83]. Although the role of the *YABBY* genes in plant evolutionary adaptation to light perception is established and they have been systematically studied in model and nonmodel species [27, 84–87], up to now, no *YABBY* genes have been described in mycoheterotrophic plants. It was interesting to figure out, if these genes and, therefore, the conserved mechanism of leaf polarity determination, were exposed to the adaptive deevolution in leafless mycoheterotroph *M. hypopitys*. Therefore, in this study we focused on the diversity and expression profile of the *YABBY* genes in a *M. hypopitys* that may further the understanding of the development and evolution of this plant group.

**Figure 7:**
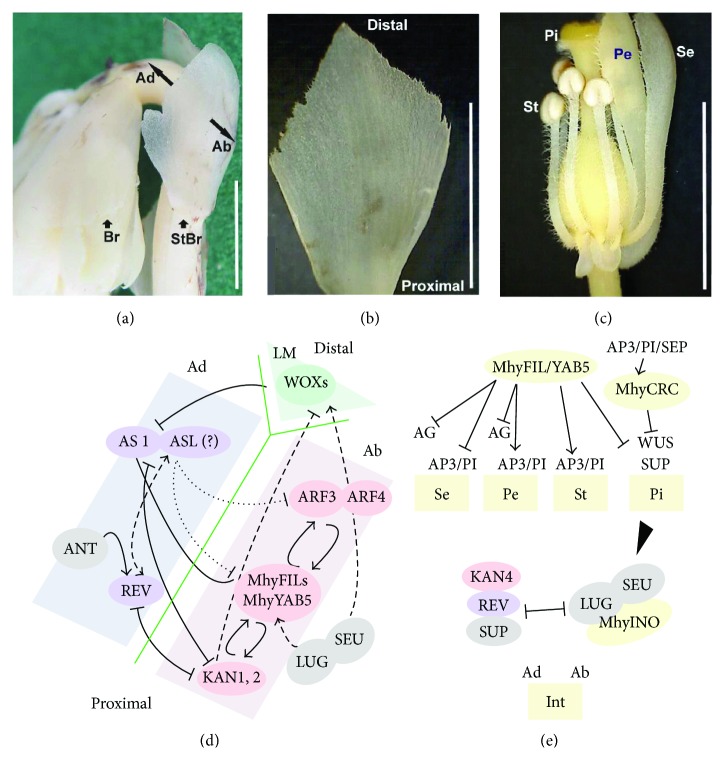
A hypothetical scenario for the functioning of the MhyYABBYs during the development of *M. hypopitys* bracts and flowers. (a) *M. hypopitys* raceme. (b) *M. hypopitys* sterile bract. (c) *M. hypopitys* flower with a partially removed perianth. (d) Scheme of possible relations between “abaxial” and “adaxial” factors in the *M. hypopitys* bracts. (e) Scheme of possible interactions of MhyYABBYs in the *M. hypopitys* flowers. Br—bract, StBr—sterile bract, Ad—adaxial side, Ab—abaxial side, LM—leaf margin, Se—sepal, Pe—petal, St—stamen, Pi—pistil, Int—integument. Scale bar = 0.5 cm.

Peripheral cells of the shoot apical meristem give rise to the leaves that develop along three axes and acquire the adaxial-abaxial and proximal-distal asymmetry and the mediolateral symmetry [88]. The elongated *M. hypopitys* raceme carries bracts below flowers and leaf-like sterile bracts [4]. Bracts are thin, 8–15 mm long, 3–15 mm broad, ovate, and expanding to the top, with irregularly toothed margins [4], rudimentary midvein, and parallel veins of similar thickness (Figures 7(a) and 7(b)). Interestingly, sterile bracts are not exactly leaves as they have the genetic signatures of reproductive organs, which are manifested in the expression of the floral organ identity MADS-box genes [89] (Figure 2).

The lack of leaves in *M. hypopitys* may correlate with possible changes in the conserved genetic network of lateral organ polarity. In pinesap transcriptomes, we found the number of genes associated with this network, including seven *YABBY*-like sequences encoding proteins, which contain conserved domains and nuclear localization signals characteristic for YABBY transcription factors.

Initially, “adaxial” and “abaxial” genes are expressed throughout the leaf primordium, and, as the leaf develops, their expression becomes restricted to their respective domains due to the mutually exclusive actions of their protein products [90]. *YABBY*s are “abaxial” genes involved in stimulation of the cellular division during lamina outgrowth in all aboveground lateral organs, vegetative or reproductive (e.g., [25, 91–93]). *M. hypopitys* flowers do not show any visible abnormalities compared to those of other eudicots. Thence, *MhyYABBY*s may play common roles in the proper development of the floral meristem into a mature flower. However, the question arises how did the lack of leaves affect the function of the “vegetative” *MhyFIL1–3* and *MhyYAB5* genes.

In *A. thaliana* leaves and sepals, *FIL* and *YAB3* genes are upregulated by KAN1 and ARF4, and, in turn, FIL and YAB3 stimulate the expression of *ARF4*, *KAN1*, and *AS1* [92, 94, 95], and besides, in complex with LUG and SEU promote not only organ polarity, but embryonic shoot apical meristem initiation and maintenance [93]. “Abaxial” KAN represses the “adaxial” *HD-ZIPIII* genes [90, 96, 97]. At the boundary between adaxial and abaxial tissues, the FIL/YAB3 and KAN, respectively, up- and downregulate the *WOX1* and *WOX3* genes that specify redundantly lateral lamina outgrowth and leaf margin cell fate [98]. The *YAB5*, *KAN2*, *ARF3*, and *ARF4* genes are repressed by the “adaxial” AS1 and AS2 implicated in the proper leaf formation along all three axes [99–101].

During flowering, *YABBY*s are required to establish a correctly developed flower primordium through the interaction with *REV*, *KAN4*, *SEU*, *LUG*, *ANT*, *SUP*, *LFY*, and the floral homeotic MADS-box genes [28, 47, 64, 65, 91, 93]. The SEU and LUG are needed to promote and maintain the *FIL/YAB3* and *HD-ZIPIII* expression [64]. In turn, FIL in combination with ANT acts to upregulate the “adaxial” gene *PHB* and MADS-box gene *AP3* [65], and together with REV, AP1, and LFY, spatially regulates the transcription of the *SUP* and the MADS-box genes *AG*, *AP3*, and *PI* [31, 44, 47]. To maintain the polar development of the ovule outer integument, the INO interacts with SEU and LUG, but its expression is restricted by KAN4, REV, and SUP [28]. CRC, upregulated by AP3/PI/SEP, is involved in the control of radial and longitudinal gynoecium growth, carpel fusion, and nectary location, and participates in the floral meristem termination through the *WUS* repression [33, 35, 36, 102].

The finding of almost all the above-described polarity genes in the analyzed transcriptomes (Supplementary Table 2; Figure 2) suggests that the polarity of *M. hypopitys* bracts and floral organs is under the control of conserved mechanisms (Figure 7), with the exception of the absence of transcripts *PHB* and *PHV* (HD-ZIPIII), *WOX1* and *WOX3* (homeodomain protein), and *AS2* (LBD domain transcription factor). The *PHB* and *PHV* function may be replaced by another member of the *HD-ZIPIII* family, *REV*, since these three genes can function redundantly [90]. Similarly, the *MhyWOX* genes may perform the functions of *WOX1* and *WOX3* [103]. Although 12 *AS2*-*like (ASL)* genes were found in *M. hypopitys* transcriptomes, the AS2 cannot be functionally replaced by other family members [104]. The lack of *AS2* transcripts may contribute to a high level of *MhyYAB5* expression compared to other “vegetative” *MhyYABBY*s, since the AS1/AS2 complex suppresses *YAB5* [88]. Moreover, the lack of *AS2* activity may be related to the characteristics of *M. hypopitys* bracts having a plump lamina base, the midvein indistinguishable from parallel veins, and the absence of petioles. This conclusion may be supported by the phenotype of the mutant *as2*, which has a significantly rudimentary leaf midvein (and several parallel veins of very similar thickness), shortened petioles and leaf blades, and a plump and swelled leaf lamina base [105].

Most of the genes that define organ polarity existed before the emergence of a flat leaf in seed plants. Based on the analyses of the families of organ polarity genes, such as *HD-ZIPIII* [106], *ARF*s [23], and *ASL*s [107], it is assumed that after the ferns' divergence, multiple paralogs arose in the seed-plant common ancestor [23]. Unlike other polarity genes, *YABBY*s originated in the lineage leading to seed plants, and it is proposed that they are implicated in the transition of an ancestral shoot-specific network into a leaf-specific one [19, 25]. At least four gene duplication events in the *YABBY* family led to the emergence of at least five *YABBY* genes with both novel and redundant functions in the last common ancestor of extant flowering plants [23, 29].

The structural and phylogenetic analysis based on comparison with the YABBY orthologs revealed that each MhyYABBY belonged to one of the four highly conserved clades in the angiosperm YABBY family [94]. In the dendrogram, it is possible to single out a cluster consisting of the FIL/YAB3, YAB5, and YAB2 clades (Figure 3). CRC- and INO-orthologs have formed separate clusters, which corresponds to the previously proposed origin of the *CRC* and *INO* genes from different ancestors [20, 23]. The tree composition was not completely congruent with the data of other studies with observations of the two clusters CRC/FIL and YAB5/YAB2/INO [23], or the two clusters FIL/CRC/INO and YAB2/YAB5 [20, 29], probably due to the inherent instability of the tree topology depending on the composition of taxa and the mode of analysis.

The obtained tree was consistent with the established phylogenetic relationships among higher plants. The presence of the MhyYABBY paralogs, which are coorthologous to the FIL and INO clades, indicates that the *MhyFIL1*/*MhyFIL2*/*MhyFIL3* and *MhyINO1*/*MhyINO2* groups could represent allelic variants (for *FIL* group), alternative splicing variants (for *INO* group), or may have originated as a result of a recent gene duplication event unique to the Ericales order.

The simplest explanation of the absence of the *YABBY2*-like transcripts in all analyzed pinesap transcriptomes may be the low abundance and insufficient transcriptome size. It is also possible that *YAB2* homologous genes are expressed at the earlier developmental stages during the formation of lateral organ primordia, which were not analyzed. One of three possible evolutionary scenarios explaining the *YABBY*s' diversification suggests that *YAB2* is the result of the earliest duplication of the *YABBY* ancestor gene [20], and therefore, can be associated with the evolution of a leaf stronger than other *YABBY*s. The *YAB2* ortholog is present in photosynthetic Ericaceae relative species *Vaccinum corymbosum*. Hence, the absence of the *YAB2* gene in *M. hypopitys* may be due to the loss of the gene during the adaptive evolution (deevolution of the genome) of the autotrophic ancestor of *M. hypopitys* accompanied by the loss of the leaf. MhyYAB2 functions could be partially complemented through neofunctionalization of MhyYAB5 or MhyFIL paralogs. Studies in *Oryza sativa* and other plants suggest that within the multicomponent regulatory network composed of homo- and heterodimers formed by “vegetative” FIL/YAB3, YAB2, and YAB5 orthologs, protein substitutions and replacements are possible [29, 108]. It was shown that in *O. sativa* the loss of the *YAB5* genes was complemented by the *FIL* and *YAB2* paralogs [108]. Accordingly, *M. hypopitys MhyYAB2* could have been replaced by the *MhyYAB5* or the three *MhyFIL*s. It is also known that YAB2 and YAB5 are important for laminar style and filament morphology evolution in angiosperms, when *YAB2* expression over a certain threshold disturbs the balance in the regulatory network, leading to radialization of the laminar structure [26, 27]. Therefore, given the *M. hypopitys* style and filament radial structure and the expression of the *MhyYAB5* gene in flower tissue, the absence of *MhyYAB2* transcripts may also indicate a possible substitution of YAB2 by YAB5 in *M. hypopitys*.

Bioinformatics analysis of the MhyYABBY structural organization revealed the presence of 18 previously known conserved motifs [29] and 7 putative novel candidate sequences for clade-specific motifs (Figure 5), which may be used as markers to identify appropriate genes. Given the shared evolutionary history of YABBYs, the novel motifs could be biologically relevant and involved in subfamily-specific functions, which need further investigation.

The *MhyYABBY* gene orthology data are supported by the *MhyYABBY* expression patterns (Figure 6). In *Arabidopsis*, “vegetative” genes *FIL*, *YAB3*, and *YAB5* are expressed in leaves and leaf-like cotyledons, sepals, petals, stamens, and carpels, whereas expression of *CRC* and *INO* is restricted to specific floral organs that are evolutionarily derived from leaves [20]. *MhyYABBY* transcripts were also found in aboveground tissues (bracts and flowers). In the case of the *MhyYAB5* gene, its atypical expression in roots indicates that it may have some roles in the development of the *M. hypopitys* root system. On the other hand, the perennial plant *M. hypopitys* commonly develops underground adventitious buds on the roots, which presumably contain an embryonic inflorescence [109], and, thus, the *MhyYAB5* gene can be expressed in the buds. Interestingly, in bracts, the expression level of *MhyYAB5* is much higher than that of *MhyFIL*s. Given the possible synergy of their action, it can be assumed that the low expression of *MhyFIL*s was compensated by an increase in the expression of the *MhyYAB5* gene not only in bracts, but also in roots, or rather in adventitious buds.

The *MhyFIL* expression profiles indicate the possible subfunctionalization of the paralogs. The *MhyFIL3* gene, which according to phylogenetic analysis is at the base of the *M. hypopitys FIL* clade, is expressed approximately at the same level as *MhyFIL1*, while the extremely low number of transcripts of the third paralog *MhyFIL2* is present only in two of the six transcriptomes (Supplementary Table 3). It is possible that *MhyFIL3* and *MhyFIL1* may have redundant functions, and *MhyFIL2* may be a pseudogene. Given trace amounts of *MhyFIL2* transcripts in the root and bud library, similar to *MhyYAB5*, it is likely that *MhyFIL2* may be involved in the development of inflorescence at the early stages after bud dormancy release [110].

The *MhyCRC* expression was detected only in flower tissue, confirming its potentially conserved roles in carpel fusion, style/stigma and nectary development, and in the floral meristem termination as it was shown for *A. thaliana* CRC [33, 38, 102], as well as in vascular development, as indicated by a recent report on the functional role of *Pisum sativum* CRC [111]. Similar to *A. thaliana* INO [40, 112], MhyINO1 and MhyINO2 may redundantly define and promote the outer ovule integument growth in *M. hypopitys*, while MhyFIL1/2/3 and MhyYAB5 may influence the abaxial cell fate in all aboveground lateral organs like their corresponding YAB1/3 and YAB5 orthologs [23].

It has recently been shown that “vegetative” YABBYs act as transcriptional activators of jasmonate-triggered responses. Jasmonate-induced degradation releases YABBYs from complexes with JAZ3 to mediate anthocyanin accumulation and chlorophyll breakdown [113]. The analysis of *M. hypopitys* transcriptome data did not reveal *JAZ3*-like transcripts, which may be consistent with complete chlorophyll loss in *M. hypopitys*. Thus, such mechanism may become evolutionarily obsolete in mycoheterotrophic plants.

The current study is the first to identify the *YABBY* genes in a mycoheterotrophic plant devoid of vegetative leaf-like organs. Seven *MhyYABBY* members were detected and classified in *M. hypopitys*, and putative protein structure, conserved motifs, and phylogenetic relationship were systematically analyzed. *MhyYABBY* transcription profiling in different plant tissues indicated the involvement of MhyYABBY proteins in the regulatory network controlling bract and flower formation. Our findings should further the investigation of YABBY functional roles in the regulation of developmental and physiological processes in achlorophyllous plant species and help to reveal possible differences in generally conserved molecular mechanisms underlying plant development and evolution.

## Abbreviations

CRC: CRABS CLAW
FIL: FILAMENTOUS FLOWER
HMG: High Mobility Group
INO: INNER NO OUTER
MhyYABBY: *M. hypopitys* YABBY
YAB: YABBY
qRT-PCR: Quantitative RT-PCR.

## Supplementary Materials

Supplementary 1 Tables 1–7: primers used for qRT-PCR analysis of pinesap gene expression (1); assembled transcripts homologous to the known organ polarity genes in six pinesap transcriptomes (2); MhyYABBY characteristics (3); estimates of evolutionary divergence between nucleotide sequences of the MhyYABBY genes (4); estimates of evolutionary divergence between amino acid sequences of the MhyYABBY proteins (5); set of YABBY orthologues from different plant species used for MEME-mediated identification of clade-specific conserved motifs (6); the significance (*p*-value) of qRT-PCR results for one gene expression between pinesap tissues (7) (in Microsoft Excel worksheet file (.xls)).

Supplementary 2 Figures 1-2: structural analysis of the *MhyYABBY* genes and encoded proteins. (1) Alignment of *M. hypopitys YABBY* genes (a) and encoded putative proteins (b); (2) novel amino acid motifs predicted in the sequence of the MhyYABBY proteins. (a)–(g) Logos created from aligned sequences and copied directly from MEME graphically represent amino acid conservation: FIL-H (a); FIL-I (b); YAB5-Cm (c); CRC-E (d); INO-B (e); INO-C (f); and INO-D (g). The height of the letters in each stack indicates the relative frequency of individual residues at the position (in portable document format (.pdf)).
