# Supplementary material for: The YABBY Genes of Leaf and Leaf-Like Organ Polarity in Leafless Plant Monotropa hypopitys
Source: Int J Genomics. 2018 Apr 24;2018:7203469. doi: 10.1155/2018/7203469 (PMC5941816; doi:10.1155/2018/7203469)

The *YABBY* genes of leaf and leaf-like organ polarity in leafless plant *Monotropa hypopitys*

Anna V. Shchennikova[1,*], Marya A. Slugina[1,2], Alexey V. Beletsky[1], Mikhail A. Filyushin[1], Andrey A. Mardanov[1], Olga A. Shulga[1], Elena Z. Kochieva[1,2], Nikolay V. Ravin[1], Konstantin G. Skryabin[1,2]

[1] Federal State Institution "Federal Research Centre "Fundamentals of Biotechnology" of the Russian Academy of Sciences", Moscow 119071, Russia

[2] Lomonosov Moscow State University, Moscow 119991, Russia

[*]Corresponding author: e-mail: shchennikova@yandex.ru

Structural analysis of the *MhyYABBY* genes and encoded proteins.

Supplementary Figure 1: Alignment of *M. hypopitys YABBY* genes (a) and encoded putative proteins (b).

Supplementary Figure 2: Novel amino acid motifs predicted in the sequence of the MhyYABBY proteins. a–g – Logos created from aligned sequences and copied directly from MEME graphically represent amino acid conservation: FIL-H (a); FIL-I (b); YAB5-Cm (c); CRC-E (d); INO-B (e); INO-C (f); INO-D (g). The height of the letters in each stack indicates relative frequency of individual residues at the position

Figure 1

a

```
                         *        20         *        40         *        60         *        80
MhyYABBY5 : ATGTCCTCCTCAAATTCTTTTTCACCGGAACACCAAACCCACCCCCACCAGCATCTCTCCCCCTCCTCCTCCG-ATCAGC :  79
MhyYABBY6 : ATGTCCTCCTCAAATTCTTATTCACCGGACCACCAAACCCACTCCCACC------TCCCCCACTCCTCCTCCG-ACCAGC :  73
MhyYABBY2 : ATGTCCTCCTCAAATTCTTATTCACCGGACCACCAAACCCACTCCCACC------TCCCCCACTCCTCCTCCG-ACCAGC :  73
MhyYABBY7 : ----------------------------------------ATGTCGAGC------TGCGCCGATGTGCCGCGGGAGCAAC :  34
MhyYABBY1 : ----------------------------------ATGGGTGATAAAGCCTCAATGGATTTGGTTCCACCAGCTGACAATC :  46
MhyYABBY3 : ----------------------------------ATGTCTAACACTACACCGAACCGAGTCTTGGACCTTCAAGAACAGA :  46
MhyYABBY4 : ----------------------------------ATGTCTAACACTACACCGAACCGAGTCTTGGACCTTCAAGAACAGA :  46
                                              a     a   c ac            c      C  c   A cA

                         *       100         *       120         *       140         *       160
MhyYABBY5 : TCTGTTACGCTCATTGCGAATTCTGCAACACTGTCCTCGTGGTGAGCGTGCCTTGCTCAAGCTTGTTCAAGACTGCGACG : 159
MhyYABBY6 : TCTGTTACGTCCAATGCAACATCTGTGACACTGTCCTCGCGGTGAGCGTGCCTTGCTCAAGCTTGTTCAAGACTGCGACG : 153
MhyYABBY2 : TCTGTTACGTCCAATGCAACATCTGTGACACTGTCCTCGCGGTGAGCGTGCCTTGCTCAAGCTTGTTCAAGGCTGTGACG : 153
MhyYABBY7 : TTTGCTACATACCTTGCAATTTTTGCAACATAGTTCTTGCGGTGAGTGTGCCATGCAGTAGCTTATTTGACACAGTTACC : 114
MhyYABBY1 : TATGCTATGTCCGTTGCAACTTCTGCAACACTGTTCTTGCGGTTGGGATACCATGCAAAAGGTTGCTGGATACAGTGACA : 126
MhyYABBY3 : TCTGCTACGTGCAATGCGGTTATTGCACTACCATTTTACTGGTAGGTGTGCCATGTGGTAGTTTGACAATGGTGGTTACA : 126
MhyYABBY4 : TCTGCTACGTGCAATGCGGTTATTGCACTACCATTTTACTGGTAGGTGTGCCATGTGGTAGTTTGACAATGGTGGTTACA : 126
            T TG TAcgt C  TGC      TG    Ac  T  T   GGT  G gTgCC TG    AG TTg          G  AC

                         *       180         *       200         *       220         *       240
MhyYABBY5 : GTTCGATGCGGCCACTGCACCAGTCTCTTTTCTGTGAACATTCGTGGCCTTCTTCTTCCTCAACCTAACCAACTCCATCA : 239
MhyYABBY6 : GTTCGATGCGGCCACTGCACCAGTCTCTTTTCTGTGAACATTCGTGGCCTTCTTCTTCCTCAACCTAACCAACTCCATCA : 233
MhyYABBY2 : GTTCGATGCGGCCACTGCACCAGTCTCTTGTCTGTGACCATGCCTGGCCTCCTTCTTCCTCAACCTAATCAGCTCCATCA : 233
MhyYABBY7 : GTCCGATGCGGGCACTGCACCAATCTGTGGTCCGTCAACAT----GGCAGCTGCCTTCC-------AATCACTCCAATCC : 183
MhyYABBY1 : GTGAAATGCGGCCATTGCAGTAACCTCTCCTTTCTCAGCAC--------------------------AAGACCCCCAACA : 180
MhyYABBY3 : GTAAGATGCGGGCACTGCACCAGCCTCCTCTCTGTCAACATGTTGAAAACCTCTTTTATCCCTCTCCATCTCTTTGCTTC : 206
MhyYABBY4 : GTAAGATGCGGGCACTGCACCAGCCTCCTCTCTGTCAACATGTTGAAAACCTCTTTTATCCCTCTCCATCTCTTTGCTTC : 206
            GT  gATGCGG CAcTGCAccA  CTc   TctgT A CAt              tt          A c        t

                         *       260         *       280         *       300         *       320
MhyYABBY5 : TCTTGGCAATTCATTATTTTCCCCTCAGAATC----TTCTGGAGGAGATCCGAA-GCTCGACCCCAAACTTGTTGATCAA : 314
MhyYABBY6 : TCTTGGCAATTCATTATTTTCCCCTCAGAATC----TTCTGGAGGAGATCCGAA-GCTCGACCCCAAACTTGTTGATCAA : 308
MhyYABBY2 : TCTTGGCCACTCATTCATTTCCCCTCAGAATC----TTTTGGACGAGATCCGAA-GCTCATCCCCAAACTTGTTGATCAA : 308
MhyYABBY7 : TTTCAGGCAC-CAAACTATGGTCCTCCGGAT-----TGCAGCATTGATTTTG---GTTCATCATCCAAATGCACGAACAA : 254
MhyYABBY1 : CAAGGGCAAT----GTTTTGATCATCAAACAA----GTTTTCAGGGTTTTGGGCCATTCGGCAGTGAAATGAACAAGGGA : 252
MhyYABBY3 : TGTCAGCCAAGATCAGATGCCAAAACAAGTACCTAGTTCTTGTGAAGAAGCAGATGGCGAACATGAAAAGGCATCAGATA : 286
MhyYABBY4 : TGTCAGCCAAGATCAGATGCCAAAACAAGTACCTAGTTCTTGTGAAGAAGCAGATGGCGAACATGAAAAGGCATCAGATA : 286
            t t  Gc A         t        Ca          tt t                g     C    AA        A   A

                         *       340         *       360         *       380         *       400
MhyYABBY5 : CC---AGCCTCACCAAAGTGAACCATTCACA-GCGGTTCGAGGGTTTGATGACCTTCCTAAGCCCCCGGCGGCTAACAGA : 390
MhyYABBY6 : CC---AGCCTCACCAAAGTGAACCATTCACA-GCGGTTCGAGGGTTTGATGACCTTCCTAAGCCCCCGGCGGCTAACAGA : 384
MhyYABBY2 : TC---AGCCCCACCCAAATGAATCATTCTCATA-GCTGTGCGAGGGTTTGATGAGCTTCCTAAGCCCGCAGCTGCTAACAGA : 384
MhyYABBY7 : CA---TGACTACGCGA---CCACCGATCATA-GCAAAT-----------AACAACGAGGGAAG-----GGCTGCAAATCGA : 312
MhyYABBY1 : C----AGTCTTCTTCATCATCTTCAAGCG-A-GCCACTC----------TCCCCTAGAGCACC----TTTTGTTGTGAAA : 312
MhyYABBY3 : GACGGAGCCCATCGCTGGTCATCTCTTCGGATGACGATGAAGAAGACGTTTTAGTCCCAATGAATCATATTGTGAATAGG : 366
MhyYABBY4 : GACGGAGCCCATCGCTGGTCATCTCTTCGGATGACGATGAAGAAGACGTTTTAGTCCCAATGAATCATATTGTGAATAGG : 366
                 aG C                   tC  A G    t            t    t     a g         G  aa ag

                         *       420         *       440         *       460         *       480
MhyYABBY5 : CCTCCGGAGAAGAGACAGAGAGTCCCATCTGCTTACAACCGCTTCATCAAGGACGAGATCCAACGCATCAAAGCTGGGAA : 470
MhyYABBY6 : CCTCCGGAGAAGAGACAGAGAGTCCCATCTGCTTACAACCGCTTCATCAAGGACGAGATCCAACGCATCAAAGCTGGGAA : 464
MhyYABBY2 : CCTCCGGAGAAGAGACAGAGAGTCCCATCTGCTTACAACCGCTTCATCAAGGACGAGATCCAACGCATCAAAGCTGGGAA : 464
MhyYABBY7 : CCTCCGGAGAAGAGACAGAGAGTACCTTCTGCATATAATCAGTTTATAAAAGAAGAGATACAGAGGATCAAGGCAAATAA : 392
MhyYABBY1 : CCACCTGAGAAGAAACACAGGCTCCCATCTGCCTACAACCGATTCATGAAGGAGGAGATACAGCGCATCAAAGCGGCCAA : 392
MhyYABBY3 : CCACCAGAGAAGAGGCAGCGGGCCCCATCAGCTTACAACAACTTCATCAAAGAGGAAATCAGAAGGCTAAAGGGGAAACA : 446
MhyYABBY4 : CCACCAGAGAAGAGGCAGCGGGCCCCATCAGCTTACAACAACTTCATCAAAGAGGAAATCAGAAGGCTAAAGGGGAAACA : 446
            CC CC GAGAAGAg CAg G g cCCaTC GC TAcAAc    TTcAT AA GA GA AT     G  T AA G      A

                         *       500         *       520         *       540         *       560
MhyYABBY5 : TCCCGACATAAGTCACAGGGAGGCGTTCAGTGCCGCTGCAAAGAATTGGGCCC---ACTTCCCCCACATTCATTTCGGAC : 547
MhyYABBY6 : TCCCGACATAAGTCACAGGGAGGCGTTCAGTGCCGCTGCAAAGAACTGGGCCC---ACTTCCCACACATTCATTTCGGGC : 541
MhyYABBY2 : TCCCGACATAAGCCACAGGGAGGCGTTCAGTGCCGCTGCAAAGAACTGGGCCC---ACTTCCCACACATTCATTTCGGGC : 541
MhyYABBY7 : TCCAGATATCAGCCATAGGGAAGCATTCAGCACTGCTGCCAAAAATTGGGCAC---ACTTCCCCCATATTCATTTTGGGC : 469
MhyYABBY1 : TCCTGAGATACCCATCGCGAAGCCCTTAGCGCTGCAGCAAAAAATTGGGCTAGGTACATTCACAAATTCACCAGCTGGGT : 472
MhyYABBY3 : TCCCAGCATGACTCACAAGGAAGCCTTCAGTGCTGCTGCAAAAAAATTGGGCCC---ATTTTCCACCAATTCAGTATAA-- : 521
MhyYABBY4 : TCCCAGCATGACTCACAAGGAAGCCTTCAGTGCTGCTGCAAAAAAATTGGGCCC---ATTTTCCACCAATTCAGTATAA-- : 521
            TCC    AT a  CA a gGA GC TTcAG gC GCtGCaAA AA TGGGC c   A tT CC c  attCa t

                         *       580         *       600         *       620         *       640
MhyYABBY5 : TCTTGCCTGAT-CAACCCCTGAAG--AAAGCTAATGTGCGCCA--GCAGGAAGGAGATGATGTGATGATGAAAGATGGGT : 622
MhyYABBY6 : TCTTGCCTGAT-CAACCGATGAAA--AAAGCTAATGTGCGCCA--GCAGGAAGGAGATGATGTGATGATGAAAGATGGGT : 616
MhyYABBY2 : TCTTGCCTGAT-CAACCGATGAAA--AAAGCTAATGTGCGCCA--GCAGGAAGGAGATGATGTGATGATGAAAGATGGGT : 616
MhyYABBY7 : TAATGCTGGAGACCACCAACCAAACTAAACTTGATGAGGTCTACTGCACAAATGAAAT----TGTTGGTCTTCAGTTGAA : 545
MhyYABBY1 : CAATGTCCAGGAGCAGTAACCATGACTGA-------------------------------------------------- : 501
MhyYABBY3 : -AAAGGGAGAAGTTAGCTGTGGTGAGGGAGCAGATCATGCAAAG-ATGTCAACGAACTGTTCAGATGCTGGAGGAGAGGT : 599
MhyYABBY4 : -AAAGGGAGAAGTTAGCTGTGGTGAGGGAGCAGATCATGCAAAG-ATGTCAACGAACTGTTCAGATGCTGGAGGAGAGGT : 599
                G   ga    A c           A    at        a        aa ga   t      g tg t          g

                         *       660         *       680         *       700         *
MhyYABBY5 : TTCTTGCTCCTGCCAATGTGGGTGTGTCCCCCTACTAA----------------------------------- : 660
MhyYABBY6 : TTCTTGGTCCTGCCAATGTGGGGGTCTCCCCCTACTAA----------------------------------- : 654
MhyYABBY2 : TTCTTGGTCCTGCCAATGTGGGGGTCTCCCCCTACTAA----------------------------------- : 654
MhyYABBY7 : ATCTCCATTGTGGAATTGA------------------------------------------------------ : 564
MhyYABBY1 : ------------------------------------------------------------------------- :   -
MhyYABBY3 : TTATGTACAAGGCAATGGATTCCATGAAAGAAAGGCTCACGCATTTTATCAGGA--CAAGGACACCCGTTGA- : 669
MhyYABBY4 : AATCTTGACTCGGTATAGTGGAAACGAAATAAAATGTGACAAGTTGGTTCCTGTTTCAATTCCAATCCGGACT : 672
                       g  a  g
```

b

```
                     *        20         *        40         *        60         *        80
MhyYABBY5 : MSSSNSFSPEHQTHPHQHLSPSSSDQLCYAHCEFCNTVLVVSVPCSSLFKTATVRCGHCTSLFSVNIRGLLLPQPNQLHH :  80
MhyYABBY6 : MSSSNSYSPDHQTHSHLPHS--SSDQLCYVQCNICDTVLAVSVPCSSLFKTATVRCGHCTSLFSVNIRGLLLPQPNQLHH :  78
MhyYABBY2 : MSSSNSYSPDHQTHSHLPHS--SSDQLCYVQCNICDTVLAVSVPCSSLFKAVTVRCGHCTSLLSVTMPGLLLPQPNQLHH :  78
MhyYABBY7 : ----------MSSCADVPRE-----QLCYIPCNFCNIVLAVSVPCSSLFDTVTVRCGHCTNLWSVNMAAAFQS----LQS :  61
MhyYABBY1 : -------MGDKASMDLVPPA----DNLCYVRCNFCNTVLAVGIPCKRLLDTVTVKCGHCSNLSFLSTRPPTQGQ-CFDHQ :  68
MhyYABBY3 : -------MSNTTPNRVLDLQ----EQICYVQCGYCTTILLVGVPCGSLTMVVTVRCGHCTSLLSVNMLKTSFIP---LHL :  66
MhyYABBY4 : -------MSNTTPNRVLDLQ----EQICYVQCGYCTTILLVGVPCGSLTMVVTVRCGHCTSLLSVNMLKTSFIP---LHL :  66
                                     q6CY  C  C t6L V 6PC sL    TV4CGHC3 L s6            lh

                     *       100         *       120         *       140         *       160
MhyYABBY5 : LGNSLFS--PQNLLEEIRSSTPNLLINQPHQSEPFTAVRGFDDL----PKPPAANRPPEKRQRVPSAYNRFIKDEIQRIK : 154
MhyYABBY6 : LGNSLFS--PQNLLEEIRSSTPNLLINQPHQSEPFTAVRGFDDL----PKPPAANRPPEKRQRVPSAYNRFIKDEIQRIK : 152
MhyYABBY2 : LGHSFIS--PQNLLDEIRSSSPNLLINQPHPNESFSAVRGFDEL----PKPAAANRPPEKRQRVPSAYNRFIKDEIQRIK : 152
MhyYABBY7 : FQAPNYG--PPDCSIDFGSSSK--CTNNMTTRPPIIAN---NNE----GR--AANRPPEKRQRVPSAYNQFIKEEIQRIK : 128
MhyYABBY1 : TSFQGFG--PFGSEMNKGQSSSSSSSEPLSPRAPFVVK------------------PPEKKHRLPSAYNRFMKEEIQRIK : 128
MhyYABBY3 : FASVSQDQMPKQVPSSCEEADGEHEKASDRRSPSLVISSDDDEEDVLVPMNHIVNRPPEKRQRAPSAYNNFIKEEIRRLK : 146
MhyYABBY4 : FASVSQDQMPKQVPSSCEEADGEHEKASDRRSPSLVISSDDDEEDVLVPMNHIVNRPPEKRQRAPSAYNNFIKEEIRRLK : 146
                     P                                            nrPPEK4qR PSAYN F6K EI R6K

                     *       180         *       200         *       220         *
MhyYABBY5 : AGNPDISHREAFSAAAKNWA-HFPHIH-------FGLLPDQPLKKANVRQQEGDDVMMKDGFLAPANVGVSPY------ : 219
MhyYABBY6 : AGNPDISHREAFSAAAKNWA-HFPHIH-------FGLLPDQPMKKANVRQQEGDDVMMKDGFLGPANVGVSPY------ : 217
MhyYABBY2 : AGNPDISHREAFSAAAKNWA-HFPHIH-------FGLLPDQPMKKANVRQQEGDDVMMKDGFLGPANVGVSPY------ : 217
MhyYABBY7 : ANNPDISHREAFSTAAKNWA-HFPHIH-------FGLMLET-----TN-QTKLDEVYCTNEIVGLQLK--SPLWN---- : 187
MhyYABBY1 : AANPEIPHREAFSAAAKNWARYIPNSP-------AGSMSRS---------SNHD------------------------- : 166
MhyYABBY3 : GKHPSMTHKEAFSAAAKNWA-HFPPIQYKKGEVSCGEGADHAKMSTNCSDAGGEVYVQGNGFHERKAHAFYQDKDTR-- : 222
MhyYABBY4 : GKHPSMTHKEAFSAAAKNWA-HFPPIQYKKGEVSCGEGADHAKMSTNCSDAGGEVILTRYSGNEIKCDKLVPVSIPIRT : 224
               P 6 H4EAFSaAAKNWA hfP i         G
```

Figure 2

a

FIL-H(11): NQP[NHS][PQA][NS][ED][SPF][LVF][MTL][PA]

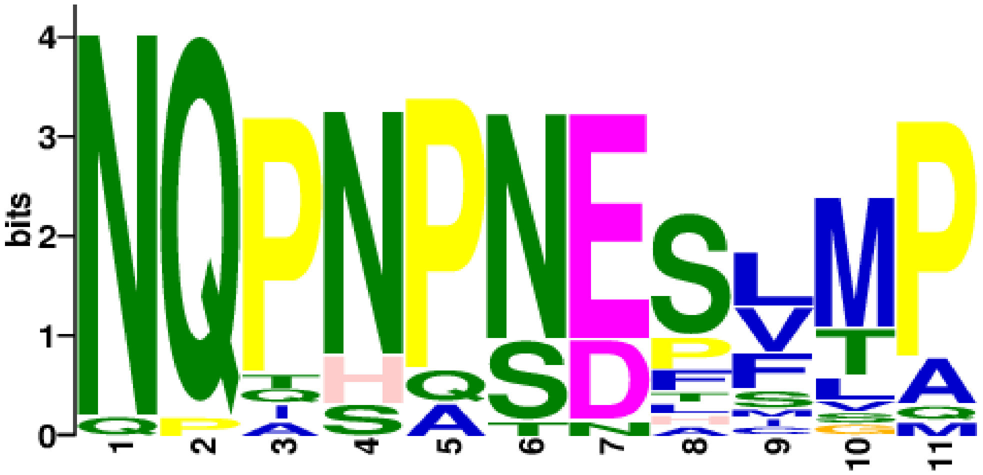

b

FIL-I(12): [GTV][VLFE][DE][ED][LI]P[KRQ]PP[VAP][AV]N

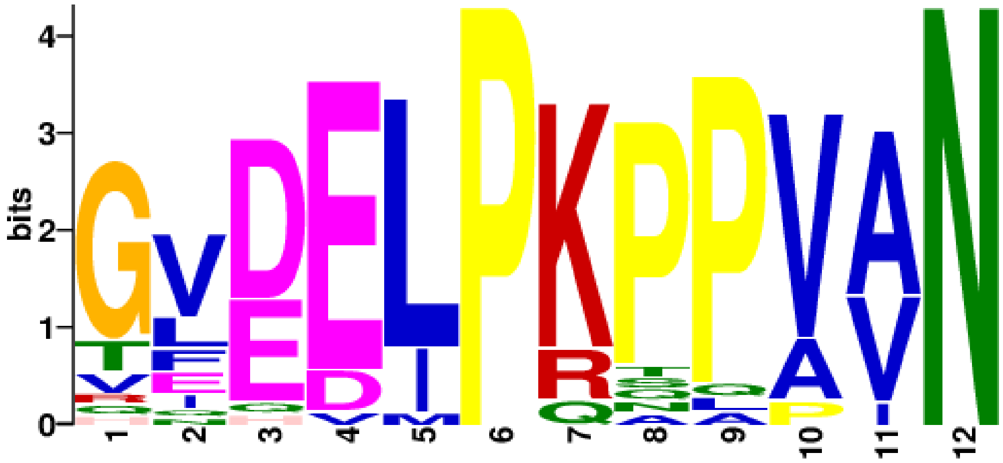

c

YAB5-Cm(12):[IPTA][ATP][NSA][NH][VTIAN][THSN]EER[VIA][VA]N

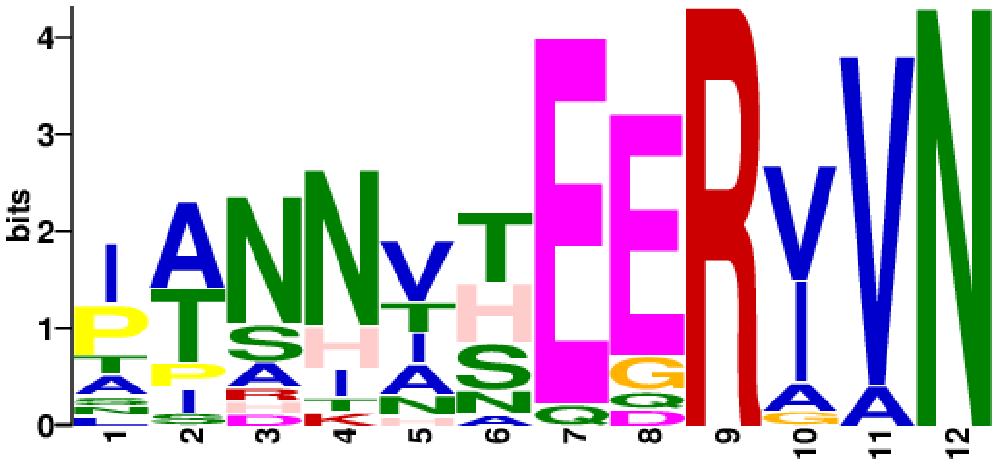

d

CRC-E(15):IP[NH][SPT]P[ATN][GS]S[VL][SA][GEA][STN][SK][NSV][NHS]

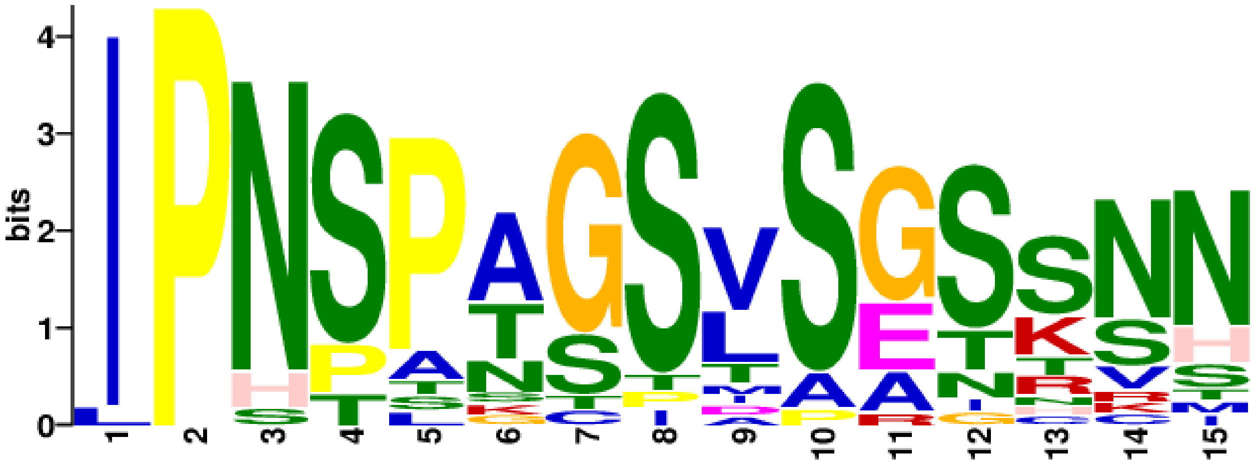

e

INO-B(11):[MN][ST][TAS]L[ND][HSR][LFY][FL][DE][LF][PQS]

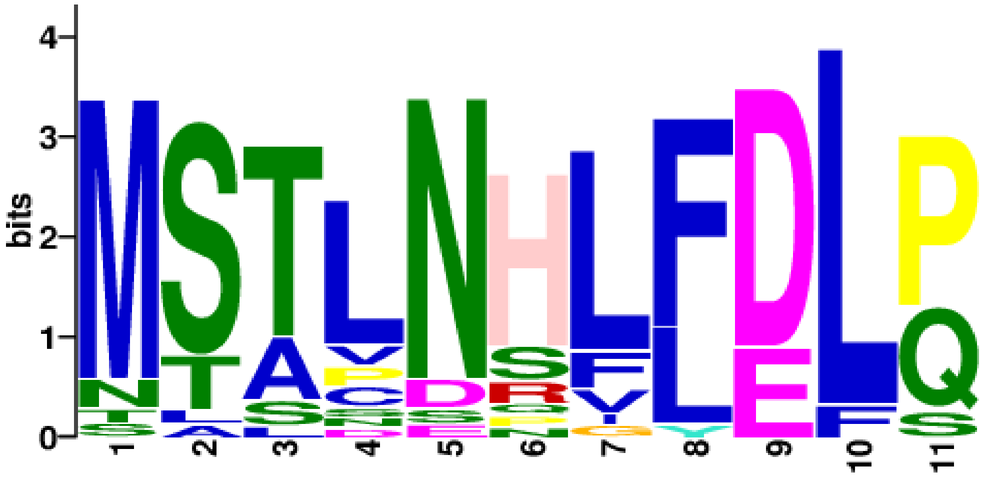

f

INO-C(15):RS[PS][STP][LMP][VM][IATFV][SY][SN][DS][NED][EDN][EDL][ED][DEY]

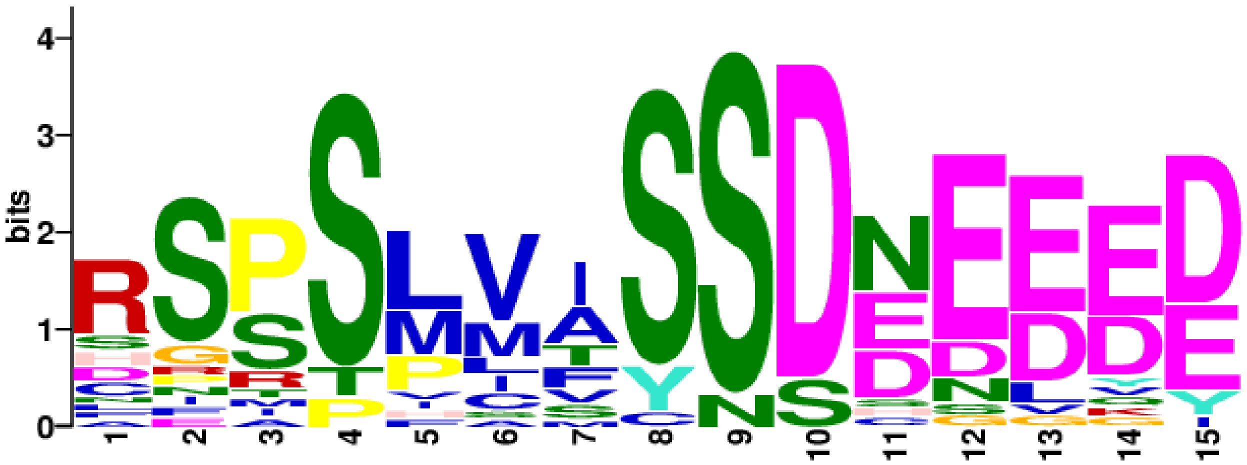

g

INO-D(26):[PG]EV[SD][KC][GD]E[GTI][DA]D[HA][NA]K[MK][SN][TS]N[SC][SD][ED][EA][GD][QG][LE][VE][NYI]

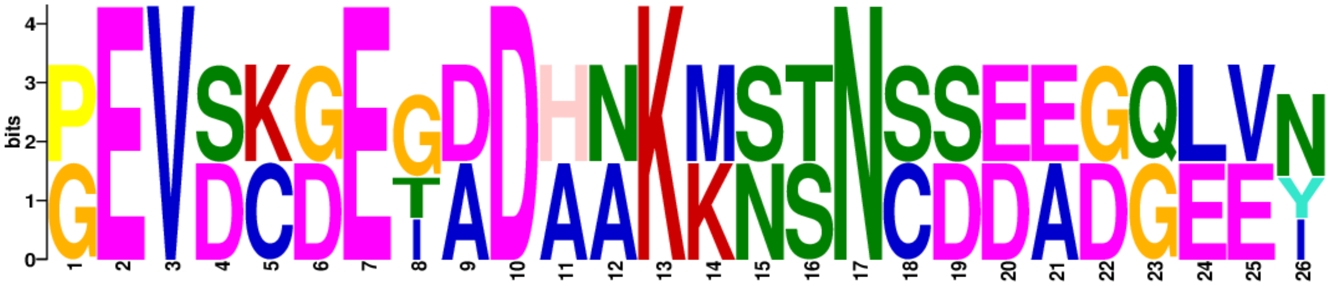

Supplement: Supplementary 2 — Figures 1-2: structural analysis of the MhyYABBY genes and encoded proteins. (1) Alignment of M. hypopitys YABBY genes (a) and encoded putative proteins (b); (2) novel amino acid motifs predicted in the sequence of the MhyYABBY proteins. (a)–(g) Logos created from aligned sequences and copied directly from MEME graphically represent amino acid conservation: FIL-H (a); FIL-I (b); YAB5-Cm (c); CRC-E (d); INO-B (e); INO-C (f); and INO-D (g). The height of the letters in each stack indicates the relative frequency of individual residues at the position (in portable document format (.pdf)). [file 7203469.f2.pdf]
